# From venom peptides to a potential diabetes treatment

**DOI:** 10.7554/eLife.44829

**Published:** 2019-02-12

**Authors:** Jiří Jiráček, Lenka Žáková

**Affiliations:** Institute of Organic Chemistry and BiochemistryCzech Academy of SciencesPrahaCzech Republic

**Keywords:** cone snail, venom, insulin, hypoglycemic shock, receptors, diabetes, Other

## Abstract

Cone snails have evolved a variety of insulin-like molecules that may help with the development of better treatments for diabetes.

**Related research article** Ahorukomeye A, Disotuar MM, Gajewiak J, Karanth S, Watkins M, Robinson SD, Salcedo P, Smith NA, Smith BJ, Schlegel A, Forbes BE, Olivera B, Chou DH-C, Safavi-Hemami H. 2019. Fish-hunting cone snail venoms are a rich source of minimized ligands of the vertebrate insulin receptor. *eLife* **8**:e41574. doi: 10.7554/eLife.41574

The hormone insulin is produced by the pancreas and is important for regulating the sugar levels in the blood. Defects in the production or function of insulin can lead to type 1 or type 2 diabetes, which affect millions of people worldwide. The lives of many of type 1 diabetics are dependent on daily insulin injections, which is also necessary for many type 2 diabetic patients (Zaykov et al., 2016).

The key to effective insulin treatment is fast-acting drugs that can quickly deliver insulin to the target cells and mimic the way that the pancreas can regulate blood sugar levels. Natural insulin is first transported to the liver, where it inhibits the synthesis of glucose, and it only reaches the peripheral tissues later, where it eventually regulates the amount of glucose entering the cells.

However, insulin injections deliver the hormone directly to the periphery, which delays the inhibition of liver glucose production and causes fluctuations of blood sugar levels (Herring et al., 2014). Moreover, at high concentrations, insulin can form aggregates that take time to dissolve and consequently contribute to delaying its action after injection (Figure 1, upper panel; Baker et al., 1988). Developing faster treatments that are not prone to aggregation is a top priority, and although several fast-acting insulins are in clinical use, they still have not reached the same pharmacodynamics as natural insulin secreted by the pancreas (Herring and Russell-Jones, 2018; Zaykov et al., 2016).

**Figure 1.**
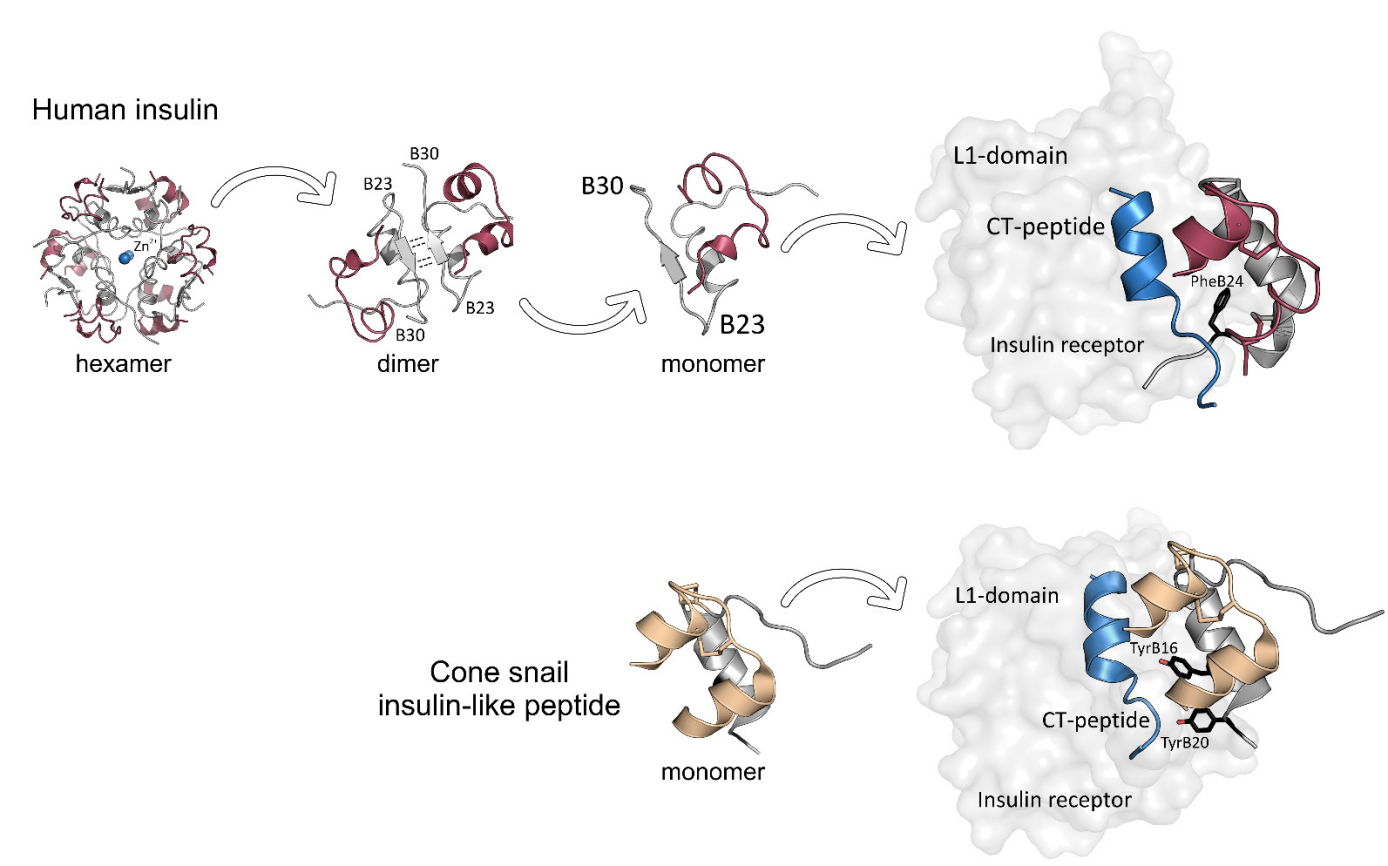
Insulin receptor binding in humans and cone snails. Upper panel: Insulin is secreted by the pancreas in the form of hexamer aggregates with ions (Zn^2+^) at their center: these aggregates divide to form dimers, which then split to form monomers. Each monomer consists of an A chain (red) and a B chain (gray) with a C-terminal B23-B30 strand. An insulin monomer binds to the L1 domain of an insulin receptor, with the C-terminus of the B chain partially detaching from the central core of the monomer to avoid a clash with the CT peptide (blue) of the receptor. A phenylalanine amino acid residue at the position B24 of insulin (PheB24, black) is crucial for insulin binding. Lower panel: Fish-hunting cone snails secrete insulin-like molecules that do not form aggregates. These molecules induce low blood sugar levels in fish, which stops them escaping. Although cone snail insulin lacks both the C-terminal of the B-chain (gray) and PheB24, it can still bind to fish insulin receptors because it contains two tyrosine amino acid residues (TyrB16 and TyrB20, black) that mimic the missing interactions. The A-chain is shown in beige.

Previous studies were able to identify the structure of the complex that insulin forms with its receptor, and the basics of how insulin binds to the receptor (De Meyts, 2015; Menting et al., 2013; Menting et al., 2014; Scapin et al., 2018; Weis et al., 2018). Now, in eLife, Helena Safavi-Hemami of the University of Utah School of Medicine and colleagues – including Peter Ahorukomeye and Maria Disotuar as joint first authors – report how cone snails could help progress our understanding of this process (Ahorukomeye et al., 2019).

Cone snails have evolved sophisticated strategies for hunting fish. They produce venomous, insulin-like molecules, which they release into the water to paralyze their victim (Figure 1, lower panel). The insulin-like molecules induce low blood sugar levels in their prey, which makes them unable to escape. It has previously been shown that the insulin-like peptide of a cone snail species does not form aggregates (Menting et al., 2016). Therefore, the researchers – who are based at institutes in the United States and Australia – wanted to find out if other cone snails also produced similar substances.

Ahorukomeye et al. discovered that although the species they analyzed made a range of different versions of these insulin-like peptides, none of the molecules could form aggregates. Moreover, the peptides were able to bind to human insulin receptors, and they were also effective in fish and mouse models of the disease, where they reversed high blood sugar levels. Structural and molecular analyses further revealed that the insulin-like peptides of the snails bind in an unusual way to the insulin receptor.

Previous research has shown that a region of insulin that is involved in the cluster formation, the C-terminus of the B chain, is also crucial for binding to the insulin receptor (Figure 1, upper panel; Baker et al., 1988; Menting et al., 2014). However, attempts to remove the B chain to avoid cluster formation have so far compromised the function of insulin.

Ahorukomeye et al. discovered that although the insulin-like peptides produced by the snails lacked the C-terminal part of the B chain, they could still effectively interact with the human insulin receptor. This might be because some their peptides contained hydrophobic amino acid residues that mimic important receptor-binding regions (e.g., phenylalanine at the position B24 of insulin) that had been lost with the missing C-terminal segment (Figure 1, lower panel). Moreover, these regions were very similar among the cone snail species studied. Taken together, these findings indicate that the C-terminus of the B chain is not necessary for insulin binding to the receptor. Future research may build on these results to design potent, fast-acting insulin agonists that are less likely to form aggregates.

The study of Ahorukomeye et al. highlights the beauty of evolution and the adaptability of organisms. We can learn important lessons from Nature as we seek to develop more patient-friendly insulin receptor agonists for the treatment of diabetes.
